# Resveratrol Improves Muscle Atrophy by Modulating Mitochondrial Quality Control in STZ‐Induced Diabetic Mice

**DOI:** 10.1002/mnfr.201700941

**Published:** 2018-04-23

**Authors:** Dongtao Wang, Huili Sun, Gaofeng Song, Yajun Yang, Xiaohu Zou, Pengxun Han, Shunmin Li

**Affiliations:** ^1^ Department of Traditional Chinese Medicine Shenzhen Hospital Southern Medical University Shenzhen Guangdong 518000 China; ^2^ Department of Nephrology Shenzhen Traditional Chinese Medicine Hospital Guangzhou University of Chinese Medicine Shenzhen Guangdong 518033 China; ^3^ Department of Nephrology Ruikang Affiliated Hospital Guangxi University of Chinese Medicine Nanning 530011 China; ^4^ Department of Pharmacology Guangdong Key Laboratory for R&D of Natural Drug Guangdong Medical College Zhanjiang 524023 China

**Keywords:** atrophy, diabetes, mitochondria, resveratrol, skeletal muscle

## Abstract

**Scope:**

In this study, we aim to determine the effects of resveratrol (RSV) on muscle atrophy in streptozocin‐induced diabetic mice and to explore mitochondrial quality control (MQC) as a possible mechanism.

**Methods and results:**

The experimental mice were fed either a control diet or an identical diet containing 0.04% RSV for 8 weeks. Examinations were subsequently carried out, including the effects of RSV on muscle atrophy and muscle function, as well as on the signaling pathways related to protein degradation and MQC processes. The results show that RSV supplementation improves muscle atrophy and muscle function, attenuates the increase in ubiquitin and muscle RING‐finger protein‐1 (MuRF‐1), and simultaneously attenuates LC3‐II and cleaved caspase‐3 in the skeletal muscle of diabetic mice. Moreover, RSV treatment of diabetic mice results in an increase in mitochondrial biogenesis and inhibition of the activation of mitophagy in skeletal muscle. RSV also protects skeletal muscle against excess mitochondrial fusion and fission in the diabetic mice.

**Conclusion:**

The results suggest that RSV ameliorates diabetes‐induced skeletal muscle atrophy by modulating MQC.

## 1 Introduction

Cachexia is a complex metabolic syndrome associated with underlying chronic diseases and is characterized by physical wasting and loss of muscle mass.^1^ It has been previously reported that the skeletal muscle is affected in patients with diabetes, leading to reduced muscle strength and physical function. Muscle atrophy in diabetes is a major threat to patient mobility and independence.^2^ Unfortunately, preventive and therapeutic interventions that arrest muscle atrophy are still at the initial stages of development.

Generally, it is believed that a direct association between skeletal muscle dysfunction and mitochondrial deficits underlies skeletal muscle atrophy. Mitochondrial homeostasis is tightly regulated by several processes, such as biogenesis, dynamics, and mitophagy, collectively referred to as mitochondrial quality control (MQC). Mitochondrial homeostasis and MQC are essential to maintenance of muscle mass and a disruption of this pathway can lead to muscle‐wasting disorders, including sarcopenia and cachexia. Mitochondrial biogenesis plays a critical role in maintaining the dynamic equilibrium of proliferation and degradation of mitochondria to meet energy demands.^3^, ^4^, ^5^, ^6^ Peroxisome proliferator‐activated receptor‐γ coactivator 1‐α (PGC1‐α) is a transcriptional regulator that upregulates mitochondrial biogenesis by inducing nuclear respiratory factors (NRF‐1 and NRF‐2) and mitochondrial transcription factor A (mtTFA) transcription, which leads to increased mitochondrial DNA replication and gene transcription.^4^, ^5^, ^7^, ^8^ Mitochondrial fusion and fission affect the distribution and quality control of mitochondria, which not only dictate the morphology of the organelle but are also responsible for maintaining mitochondrial turnover through their contributions to the biogenesis and degradation of organelles.^3^, ^9^, ^10^ Mitochondrial fission depends on the cytosolic GTPase dynamin‐related protein 1 (DRP1), which is recruited to the outer mitochondrial membrane where it assembles into multimeric ring complexes that form active GTP‐dependent mitochondrial fission sites.^11^ Interestingly, phosphorylation of different residues of DRP1 causes opposing effects. Phosphorylation at Ser616 stimulates mitochondrial fission, whereas phosphorylation at Ser637 inhibits fission.^12^ Additionally, DRP1 interacts with the integral outer mitochondrial membrane proteins, including mitochondrial fission‐1 (Fis1) and mitochondrial fission factor (MFF). In contrast, mitochondrial fusion is primarily regulated by members of the dynamin‐related GTPases, mitofusin‐2 (Mfn‐2) and optic atrophy protein‐1 (OPA1), which tether and fuse the outer and inner mitochondrial membranes, respectively.^13^, ^14^ Mitophagy was thought to be closely associated with the mitochondrial fusion and fission processes, which contribute to alterations in mitochondrial morphology and dynamics.^15^, ^16^ Thus, NIP3‐like protein X (NIX; also known as BNIP3L) outer mitochondrial membrane protein binds to LC3 on the isolated membranes, which mediates the selective recognition and targeting of damaged mitochondria by autophagosomes. Once mitochondria are damaged by losing their membrane potential, PTEN‐induced putative kinase 1 (PINK1) selectively recruits the ubiquitin ligase, Parkin, to damaged mitochondria and modifies mitochondria by polyubiquitination, leading to mitophagy. Disturbances in any of these processes may lead to defective MQC. Thus, skeletal muscle mass is tightly regulated by MQC processes and slight perturbations in these pathways can lead to muscle atrophy. However, the role of the MQC system in diabetic muscle atrophy has not been elucidated.

Resveratrol (RSV), a natural antioxidant found mainly in peanuts, pines, grape skin, and especially red wine, has been shown to inhibit protein degradation and attenuate skeletal muscle fiber atrophy in several in vitro studies.^17^, ^18^, ^19^, ^20^ Furthermore, RSV has recently been shown to improve muscle atrophy in several disease models, including diabetes, cancer, and disuse syndrome.^21^, ^22^, ^23^, ^24^, ^25^ However, the effect of RSV on MQC processes in diabetic muscle atrophy is unclear. Hence, the aim of the present study was to investigate how diabetes mellitus (DM) impairs MQC processes and subsequently contributes to muscle atrophy via activating catabolic signaling pathways. Moreover, we studied how RSV could blunt diabetic muscle atrophy by modulating MQC processes.

## 2 Experimental Section

### 2.1 Animals and Procedures

The experimental and feeding protocols were in accordance with the National Health guidelines and were approved by the Guangzhou University of Chinese Medicine Institutional Animal Care and Use Committee (SYXK (Yue) 2017‐0179). C57BL/6 mice (male), 6 to 7 weeks old, were purchased from Guangdong Medical Laboratory Animal Center (GDMLAC), Guangzhou, China. Animals were housed at constant room temperature (20 ± 1 °C) under a controlled 12 h light, 12 h dark cycle and had free access to water and food. DM was induced in mice by intraperitoneal injection of streptozocin (STZ) (Sigma‐Aldrich, St. Louis, MO, USA; 200 mg kg^−1^ body weight) dissolved in citrate buffer (0.1 m; pH 4.2), as previously described.^26^ As a control (CTL), only citrate buffer was injected. Blood glucose concentration was assessed using a glucometer (Roche, Basel, Switzerland) from the tail vein after 3 d to confirm that glucose levels greater than 300 mg dL^−1^ (16.7 mmol L^−1^) were reached after STZ injection. The CTL and DM mice received either a control diet of normal mouse chow (AIN‐76A Rodent Diet; GDMLAC) or an identical diet containing 0.04% (w/w) RSV (Sigma‐Aldrich; equivalent dose of 100 mg kg^−1^ d^−1^). There were four treatment groups (*n* = 10 per group): 1) CTL group receiving saline injection and normal chow, 2) RSV CTL group receiving saline injection and RSV chow, 3) DM group receiving STZ injection and normal chow, and 4) RSV group receiving STZ injection and RSV chow. During the 8‐week experiment, body weights were recorded every week. Food intake was measured using metabolic cages (Tecniplast S.p.A., Buguggiate, Italy) at 7 weeks following initiation of RSV treatment. By the end of the study, two mice in the DM group had died, but none had died in other groups.

### 2.2 Biochemical Parameters

After 8 weeks of treatment, the mice were euthanized by sodium pentobarbital injection and blood samples were collected immediately. Serum biochemical indexes (blood glucose, serum creatinine (SCr), blood urea nitrogen (BUN), serum albumin (ALB), alanine transaminase (ALT), and aspartate transaminase (AST)) were detected using a Roche Automatic Biochemical Analyzer.

### 2.3 Grip Strength and Running Distance

Grip strength was measured using a dynamometer for mice (ZH‐YLS‐13A, Anhui Zhenghua Biological Instrument Equipment Co. Ltd., Huaibei, China). Briefly, for the forelimb testing procedure, the mice were allowed to engage the grip and were then pulled backward until they released. For the hindlimbs, the mice were started in the middle of the platform and were pulled backward across the acrylic plate, engaging the grip. The mice continued to be pulled backward until they released. The PC interface software automatically sensed compression or tension and recorded the peak value (in N) with the mouse identification number previously entered into the software. The gauge automatically reset to zero after each measurement.

The running distance was measured using a treadmill for mice (Yuyan Instrument Equipment Co. Ltd., Shanghai, China). Briefly, the mice were acclimated to the treadmill running for 5 min at a speed of 10 m min^−1^ on a 0% grade. After acclimation, the mice were run on the treadmill with a 10% uphill grade starting at a speed of 10 m min^−1^ for 5 min. Every 2 min, the speed was increased by 2 m min^−1^ until the mice were exhausted. The running time and speeds were recorded and the running distance was calculated.

### 2.4 Morphological Studies (Histological Staining)

The muscles were photographed by digital camera and their lengths were measured by metallic measuring digital vernier caliper (Science Use Digital Vernier Caliper 150 mm Range 0.02 mm Accuracy, Foshan Songqi Technology Co. Ltd). Paraffin sections of the tibialis anterior (TA) muscles were stained with hematoxylin and eosin according to standard protocols; myofiber cross‐sectional area was then determined as we previously reported.^27^ Frozen sections of the TA muscle were stained with succinate dehydrogenase (SDH, complex II of the respiratory chain) for measurements of SDH activity and classification of fiber type as I (slow oxidative), IIa (fast oxidative glycolytic), or IIb (fast glycolytic), in accordance with our previously described protocol.^28^

### 2.5 Ultrastructural Analysis (Transmission Electron Microscopy)

The detailed procedures of transmission electron microscopy (TEM) for muscle were previously reported.^29^ Briefly, sections of TA muscle 1 mm^3^ in volume were fixed in 2.5% glutaraldehyde followed by postfixation in 1% osmium tetroxide. Image J software was used to analyze images collected by TEM (JEM‐1400, JEOL Ltd., Tokyo, Japan) under 12 000× magnification. Mitochondrial content was determined by quantifying the mitochondrial number and the size (minimum diameter) of each mitochondrion per field. A total of 20 fields per per mouse performed in 3 mice per condition were analyzed.^30^

### 2.6 Mitochondrial Isolation

The mitochondrial isolation from skeletal muscle was modified from the protocol described by Boutagy et al.^31^ Briefly, the red muscle was dissected from the quadriceps (Quad) muscle, finely minced with scissors, and then transferred to 10 mL mitochondrial homogenate buffer. The muscle was homogenized using a tissue homogenizer, keeping the homogenate on ice at all times. After homogenization, the homogenate was transferred to a 15 mL centrifuge tube and centrifuged at 1300 *g* for 5 min at 4 °C, absorbing the supernatant onto mitochondrial centrifugation buffer in a high‐speed centrifuge tube and centrifuged at 17 000 *g* for 10 min at 4 °C. The resulting pellet was resuspended in 9 mL isolation buffer in another prechilled high‐speed centrifuge tube and centrifuged at 10 000 *g* for 10 min at 4 °C. The pellet was resuspended in 1 mL isolation buffer, transferred to a new prechilled 1.5 mL microcentrifuge tube and centrifuge at 8000 *g* for 10 min at 4 °C. The pellet was gently resuspended in 300 μL storage buffer, aliquoted, and stored at –80 °C for later use.

### 2.7 Quantitative Reverse Transcription Polymerase Chain Reaction (PCR)

Total RNA was isolated from Quad muscle using TRIzol (Invitrogen, Carlsbad, CA, USA). RNA concentration and integrity were assessed using the Agilent RNA 6000 Nano Kit (Agilent Technologies, Inc., CA, USA) in an Agilent 2100 Bioanalyzer. cDNA was synthesized using iScript™ cDNA Synthesis Kit, by incubating at 70 °C for 10 min, followed by 42 °C for 60 min, and finally 95 °C for 10 min. The sequences of the specific primers are as follows: ubiquitin, forward, 5′‐TAAGACCATCCTCGATT‐3′ and reverse, 5′‐TGGATGTTGTAGTCAAGGG‐3′; GAPDH, forward, 5′‐ATGCTGGTGCTGAGTATGTC‐3′ and reverse, 5′‐AGTTGTCATATTTCTCGTGG‐3. All primers were synthesized by Invitrogen. Quantitative PCRs for all genes were run separately and amplifications were performed on the ABI Prism^®^ 5700 Sequence Detection System (Applied Biosystems) using SYBR® Green PCR Master Mix (Applied Biosystems). Results were quantified as threshold cycle (*C*_t_) values. Expressions were normalized to the corresponding GAPDH values. The levels of the sham group were arbitrarily set to 1.

### 2.8 Western Blotting

Snap‐frozen Quad muscle tissues were homogenized in lysis buffer, as we previously reported.^32^ Cytosolic and mitochondrial proteins were separated on a 10% SDS‐PAGE gel and then transferred to a PVDF membrane (Bio‐Rad Laboratories, Hercules, CA, USA). Nonspecific binding sites on the membrane were blocked at room temperature for 1 h with 5% non‐fat milk powder in Tris‐buffered saline/Tween‐20 (TBST) and then incubated overnight at 4 °C with primary antibodies. After washing with TBST, the membranes were incubated with secondary antibodies for 1 h at room temperature with shaking. After washing, the protein bands were detected and analyzed using a ChemiDoc™ MP Imaging System (Bio‐Rad Laboratories, CA, USA). Voltage‐dependent anion channel (VDAC) and GAPDH were used as loading controls for mitochondrial and cytosolic proteins, respectively. Results are expressed as the integrated optical density relative to the corresponding VDAC or GAPDH value. VDAC (1:1000, #4661), p‐DRP1(Ser616) (1:1000, #3455), Mfn‐2 (1:1000, #9482), DRP1 (1:1000, #8570), cytochrome c oxidase subunit IV (Cox IV, 1:1000, #4844), Caspase‐3 (1:1000, #14220), and Bcl2/adenovirus E1B 19 kDa protein‐interacting protein 3‐like (BNIP3L/Nix, 1:1000, #12396) antibodies were from Cell Signaling Technologies (Danvers, MA, USA). LC3 I/II (1:1000, ab58610), p‐Parkin (1:1000, ab154995), Parkin (1:1000, ab77924), and PINK1 (1:1000, ab23707) antibodies were from Abcam (Cambridge, UK). ATP synthase, H+ transporting, mitochondrial F1 complex, beta polypeptide (ATP5B, 1:1000, ARP48185_T100) antibody was from Aviva Systems Biology (San Diego, CA, USA). p‐DRP1(Ser637) (1:1000, GTX50911), MFF (1:1000, GTX64071), and MuRF‐1 (1:1000, GTX110475) antibodies were from Gene Tex (San Antonio, TX, USA). Fis1 (1:100, sc‐98900), mtTFA (1:100, sc‐376672), and NRF‐1 (1:100, sc‐33771) antibodies were from Santa Cruz Biotechnology (CA, USA). OPA1 (1:1000, 612606) was from BD Biosciences (San Jose, CA, USA). Atrogin‐1 (1:1000, AP2041) was from ECM Biosciences (Versailles, KY, USA). PGC‐1α (1:2000, NBP1‐04676) was from Novus Biologicals (Colorado, USA). GAPDH (1:1000, 60004‐1‐Ig) was from Proteintech Group, Inc. (Chicago, IL, USA).

### 2.9 Statistical Analysis

All statistical analyses were carried out by two‐way analysis of variance (ANOVA) to compare differences among multiple groups, followed by Bonferroni post hoc analysis to specifically test individual differences between groups. Statistical analyses were performed using SPSS statistical software, version 16.0. Data are presented as means ± standard deviations (SDs). Differences were considered statistically significant if *p* < 0.05.

## 3 Results

### 3.1 Changes in Food Intake, Renal Function, and Liver Function

**Figure 1:**
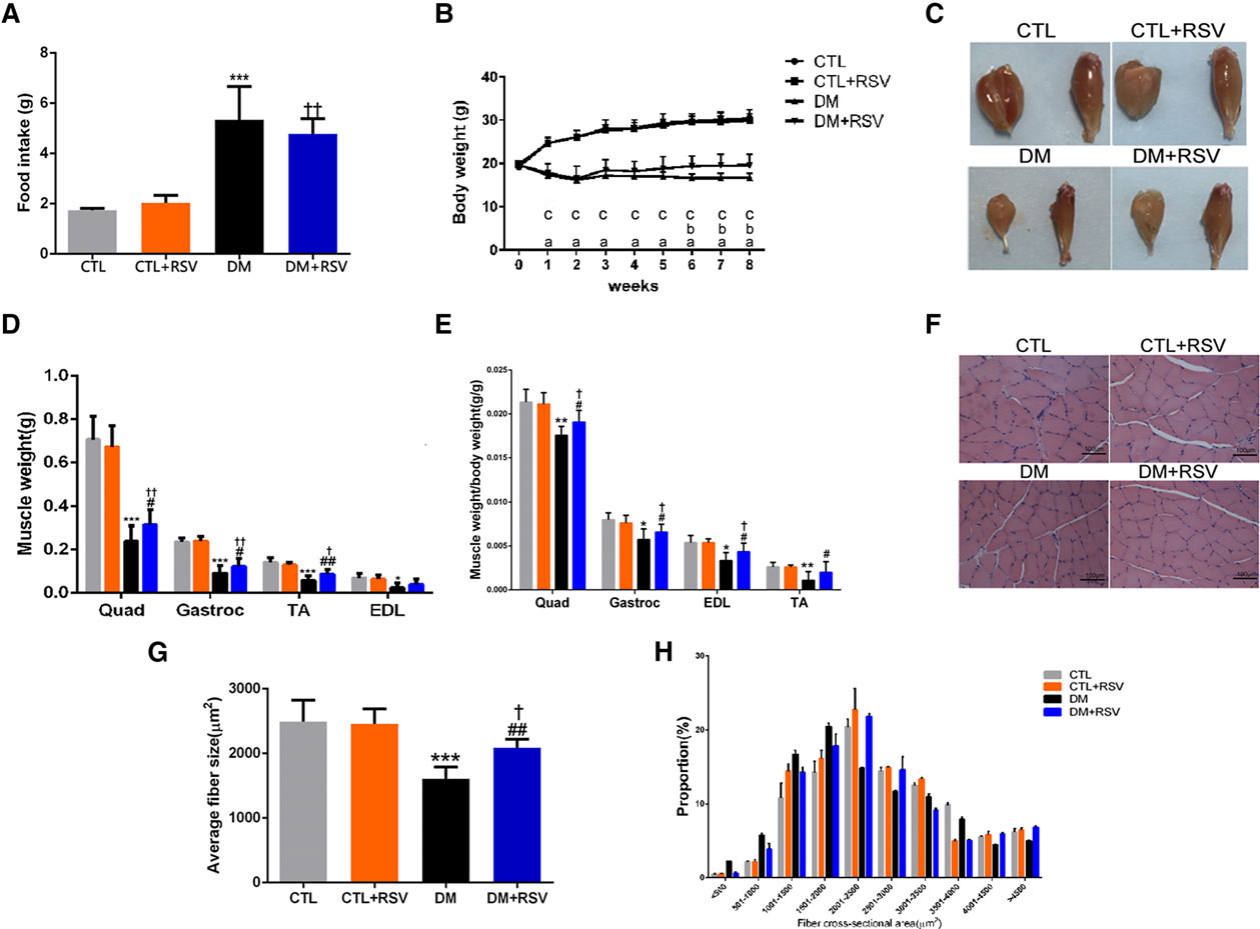
Feeding the diabetes mellitus (DM) mice with the RSV‐enriched diet preserved muscle atrophy in the model of DM induced by streptozotocin. A) Food intake. B) Body weight. C) Macroscopic view of the gastrocnemius (Gastroc) and tibialis anterior (TA) muscles. D) Weight of the quadriceps (Quad), Gastroc, TA, and extensor digitorum longus (EDL) muscles were normalized to tibia length. E) Changes in Quad, Gastroc, TA, and EDL normalized to body weight. F) Hematoxylin and Eosin (HE) staining of the TA muscle. Scale bar: 100 μm. G) Average fiber size of the HE‐stained TA muscle. H) Muscle fiber frequency distribution of the HE‐stained TA muscle. Results are presented as mean ± SD, *n* = 4–10 per group. a, *p* < 0.001 between control (CTL) and DM groups; b, *p* < 0.05 between DM and DM+RSV groups; c, *p* < 0.05 between CTL+RSV and DM+RSV groups. *p < 0.05; ***p < 0.001 between CTL and DM groups. #*p* < 0.05; ##*p* < 0.01 between DM and DM+RSV groups. ^†^ *p* < 0.05; ^††^ *p* < 0.01 between CTL+RSV and DM+RSV groups.

Compared to that of the CTL group, the DM group showed obvious increased food intake. Meanwhile, the RSV‐treated DM group displayed greater food intake than that of the RSV‐treated CTL group. However, both DM and RSV‐treated DM groups exhibited similar food intake (DM: 5.57 ± 1.31 g d^−1^ and RSV: 4.78 ± 0.50 g d^−1^) during the 8‐week experimental period (**Figure** 1A). Additionally, after the 8 weeks, the DM group displayed higher BUN and ALT and lower ALB levels compared to those of the CTL group. The levels of SCr, BUN, ALB, AST, and ALT did not differ significantly between the DM and RSV‐treated DM groups (**Table** 1).

**Table 1:** Blood glucose, renal, and liver function data (*n* = 4–7 per group)

Group	Blood glucose [mmol L^−1^]	SCr [μmol L^−1^]	BUN [mmol L^−1^]	ALB (g L^−1^)	ALT [U L^−1^]	AST [U L^−1^]
CTL	5.81 ± 1.05	12.00 ± 1.55	12.48 ± 0.57	40.90 ± 1.73	40.78 ± 8.60	151.35 ± 45.11
CTL+RSV	5.98 ± 0.98	14.00 ± 2.83	12.63 ± 1.58	41.70 ± 0.74	41.93 ± 18.67	195.33 ± 82.29
DM	25.34 ± 2.70^b^	14.67 ± 4.84	16.85 ± 4.57^a^	33.48 ± 1.93^b^	63.17 ± 21.97^a^	181.87 ± 63.13
DM+RSV	23.56 ± 3.12^d^	13.29 ± 1.89	13.94 ± 2.83	33.50 ± 3.06^d^	66.97 ± 28.70^c^	162.73 ± 44.93

Data are expressed as means ± SD

a *p* < 0.05

b *p* < 0.001 between CTL and DM groups

c *p* < 0.05

d *p* < 0.001, CTL+RSV group versus DM+RSV group.

### 3.2 Resveratrol Increases Body Weight and Muscle Mass in DM Mice

There were no differences in body weights among the groups at the beginning of the experimental protocol. However, the body weight of the DM mice gradually decreased, whereas that of the CTL and RSV‐treated CTL groups steadily increased over the 8 weeks. Compared to that of the DM group, the RSV‐treated DM group showed obvious improvement in body weight; from 6 weeks, the body weight of the RSV‐treated DM group tended to be higher than that of the DM group (Figure 1B). The increase in body weight with RSV included an increase in the weight of the Quad, gastrocnemius (Gastroc), and TA muscles in the DM group (Figure 1C and D). The weights of the Quad, Gastroc, TA, and extensor digitorum longus muscles, corrected for body weight, were significantly lower in the DM group than in the CTL group, whereas they were higher in the RSV‐treated DM group than in the DM group (Figure 1E). The improved muscle mass in the RSV‐treated DM group was confirmed by a rightward shift in the distribution of myofiber sizes (Figure 1H) and by an increase in the average cross‐sectional area of the myofibers in TA muscles (Figure 1F and G).

### 3.3 Resveratrol Improves Muscle Function in DM Mice

**Figure 2:**
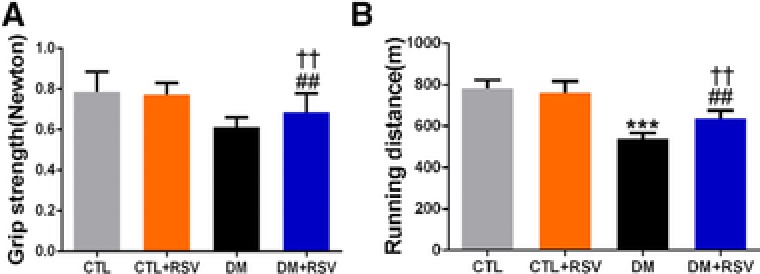
Feeding the diabetes mellitus (DM) mice with the resveratrol (RSV)‐enriched diet improved muscle function in the model of DM induced by streptozotocin. A) Grip strength. B) Running distance. Results are presented as mean ± SD, *n* = 4–10 per group; ***p < 0.001 between control (CTL) and DM groups. ##*p* < 0.01 between DM and DM+RSV groups. ^††^ *p* < 0.01 between CTL+RSV and DM+RSV groups.

Concomitant with the measurements of muscle mass, muscle function was assessed by grip strength and treadmill‐running tests. The data indicate lower grip strength and running distance in the DM group than in the CTL group. However, treatment with RSV ameliorated the reduced grip strength (**Figure** 2A) and running distance (Figure 2B) observed with DM.

### 3.4 Resveratrol Inhibits the Ubiquitin–Proteasome System, Autophagy, and Apoptosis in Skeletal Muscle of DM Mice

**Figure 3:**
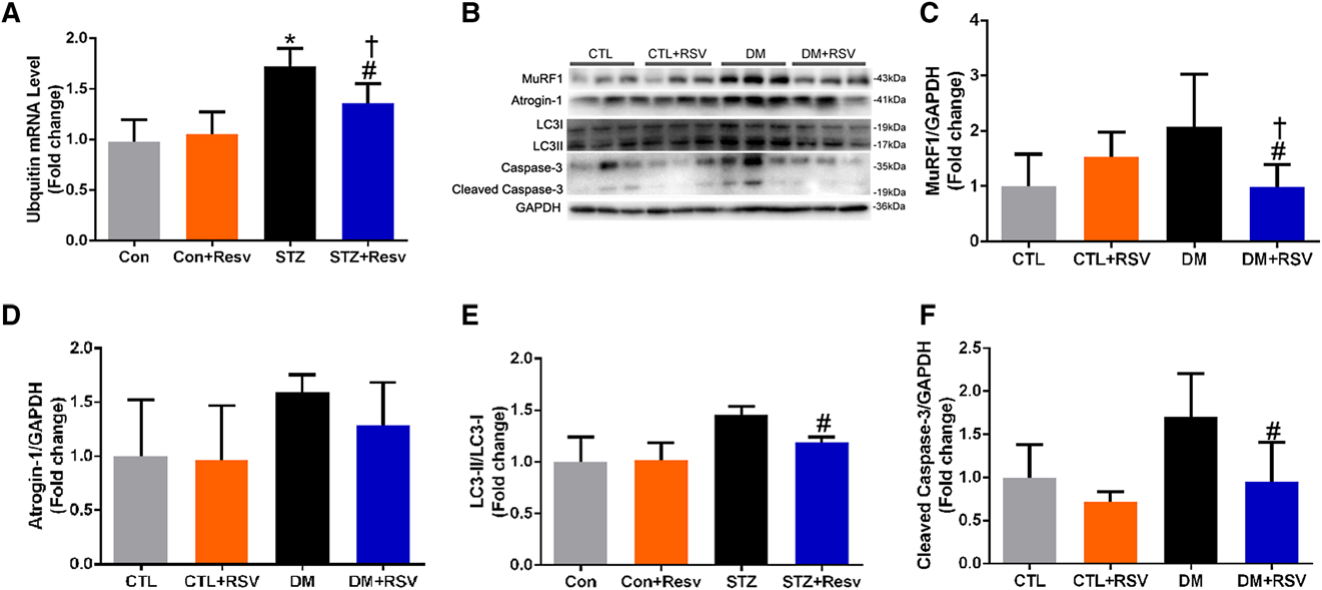
Feeding the diabetes mellitus (DM) mice with the RSV‐enriched diet prevented increased muscle atrophy markers in the quadriceps muscle. A) The expression of ubiquitin mRNA. B) Representative western blots using antibodies against MuRF‐1, atrogin‐1, LC3‐II, and caspase‐3. After quantification, the C) MuRF‐1/GAPDH, D) atrogin‐1/GAPDH, E) LC3‐II/LC3‐I, and F) cleaved caspase‐3/GAPDH ratios were calculated in mice fed either the control (CTL) or RSV‐enriched diet. GAPDH was used as a loading control. Data are expressed as fold change versus CTL and are reported as mean ± SD, *n* = 4–6 per group; *p < 0.05 between CTL and DM groups. #*p* < 0.05 between DM and DM+RSV groups. ^†^ *p* < 0.05 between CTL+RSV and DM+RSV groups.

The DM group displayed an increase in the expression of two muscle‐specific E3 ubiquitin ligases, muscle atrophy F‐box (MAFbx)/atrogin‐1 and MuRF‐1, and the increased MuRF‐1 was attenuated by RSV administration (**Figure** 3B–D). In addition, to confirm the relevance of the changes in Atrogin‐1 and MuRF‐1 seen in the DM mice, we measured ubiquitin mRNA. As shown in Figure 3A, the level of ubiquitin mRNA was upregulated in the DM group, and this was also prevented by RSV treatment. Additionally, the cleavage of caspase‐3 and the ratio of LC3‐II/LC3‐I were increased in the DM group, and these changes were abolished by feeding RSV chow (Figure 3E and F). The prevention of these changes by RSV suggests that RSV‐attenuated proteolysis is mediated at least in part through these pathways.

### 3.5 Resveratrol Improves Mitochondrial Content and Aberrant Muscle Morphological Features in Skeletal Muscle of DM Mice

**Figure 4:**
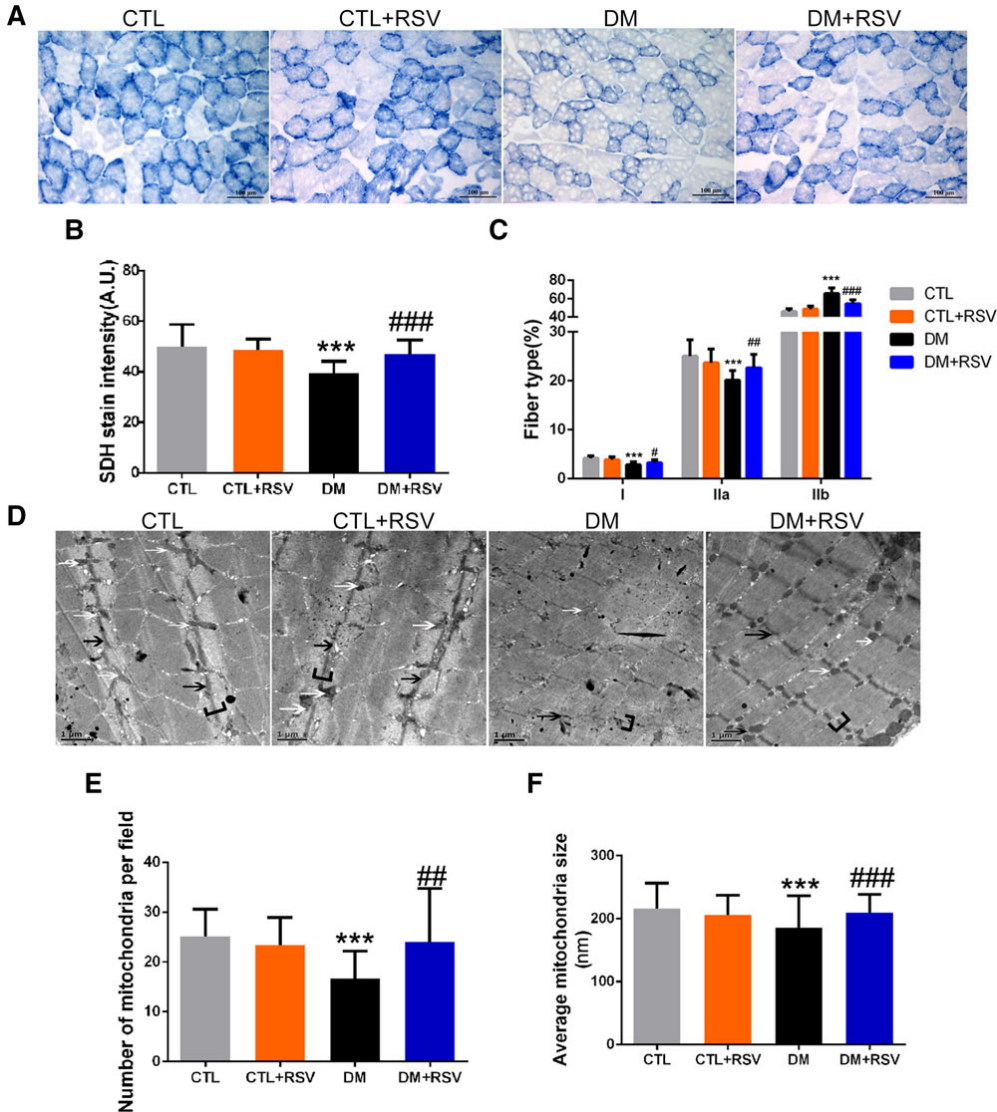
Feeding the diabetes mellitus (DM) mice with the RSV‐enriched diet increased mitochondrial content and improved aberrant muscle morphological features in the gastrocnemius (Gastroc) muscle. A) Succinate dehydrogenase (SDH) staining was performed on 10 μm‐thick sections from Gastroc muscles frozen in liquid nitrogen‐cooled isopentane. B) Quantification of succinate dehydrogenase (SDH) stain intensity (expressed in A.U.) and C) number of type I (slow oxidative), IIa (fast oxidative glycolytic) muscle fibers, and type IIb (fast glycolytic) muscle fibers. Scale bar: 100 μm. D) Transmission electron microscopy micrographs (magnification: 12 000×) of Gastroc muscles. White arrows indicate mitochondria. Black arrows indicate the Z‐line. Brackets identify the I‐bands. Scale bar: 1 μm. E) Quantification of mitochondrial amount (number per field) and F) size (average diameter, nm). Data are expressed as mean ± SD, *n* = 4 per group; ***p < 0.001 between control (CTL) and DM groups. #*p* < 0.05, ##*p* < 0.01, ###*p* < 0.001 between DM and DM+RSV groups.

Mitochondrial content was assessed using SDH staining and TEM of Gastroc muscles (**Figure** 4A and D). The SDH activity, which represents mitochondrial amount, was markedly reduced in the DM group and RSV ameliorated this response (Figure 4B). SDH staining revealed that type I (slow oxidative) and IIa (fast oxidative glycolytic) muscle fibers, which are mitochondria‐rich fibers, were significantly decreased, and type IIb (fast glycolytic) fibers were increased in the DM group. Quite interestingly, we found that RSV treatment altered the muscle fiber composition in the DM group, characterized by a reduction in type IIb fibers and a significant increase in type I and IIa fibers (Figure 4C). These results indicate that there was a substantial return to control levels in the fiber‐type distribution in the RSV‐treated DM group. Consistent with the decrease in SDH activity, the TEM morphology also revealed major alterations at the sarcomeric level, with abnormalities consistent with fewer and smaller mitochondria (white arrows; Figure 6E and F) and with markedly thinner Z‐lines (black arrows; Figure 4D) in the DM group compared to that of the CTL group. Similarly, the I‐band, mainly consisting of thin actin filaments, was thin or completely absent in the DM group (brackets; Figure 4D). Importantly, these changes were prevented by RSV treatment.

### 3.6 Resveratrol Increases Mitochondrial Biogenesis in Skeletal Muscle of DM Mice

**Figure 5:**
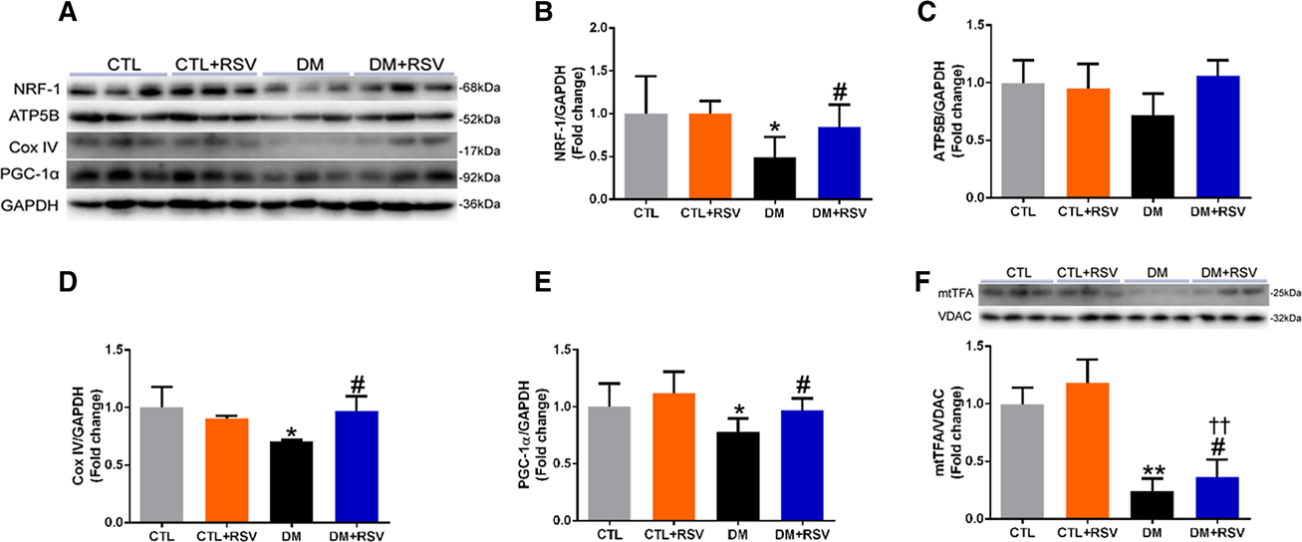
Feeding the diabetes mellitus (DM) mice with the RSV‐enriched diet improved mitochondrial biogenesis in the quadriceps muscle. A) Representative western blots using antibodies against nuclear respiratory factor (NRF‐1), ATP synthase, H+ transporting, mitochondrial F1 complex, beta polypeptide (ATP5B), cytochrome c oxidase subunit IV (Cox IV), peroxisome proliferator‐activated receptor‐γ coactivator 1‐α (PGC‐1α), and GAPDH. After quantification, the B) NRF‐1/GAPDH, C) ATP5B/GAPDH, D) Cox IV/GAPDH, and E) PGC‐1α/GAPDH ratios were calculated in mice fed either the control (CTL) or RSV‐enriched diet. GAPDH was used as a loading control. F) Representative western blots using antibodies against mitochondrial transcription factor A (mtTFA) and voltage‐dependent anion channel (VDAC), and the mtTFA/VDAC ratio was determined. The mitochondrial protein VDAC was used as a loading control. Data are expressed as fold change versus CTL and reported as mean ± SD, *n* = 4–6 per group; *p < 0.05; **p < 0.01 between CTL and DM groups. #*p* < 0.05 between DM and DM+RSV groups; ^††^ *p* < 0.05 between CTL+RSV and DM+RSV groups.

DM resulted in reduced protein expression of NRF‐1, Cox IV, and PGC‐1α and RSV significantly attenuated those changes (**Figure** 5A, B, D, and E). However, the protein levels of ATP5B were not different among groups (Figure 5C). In addition, the levels of mtTFA in the isolated muscle mitochondria of DM mice appeared to be decreased and these changes were attenuated with RSV (Figure 5F). Taken together, these results indicate that RSV antagonizes the reduction in muscle mitochondrial biogenesis in DM mice.

### 3.7 Resveratrol Inhibits Mitochondrial Fission and Fusion in Skeletal Muscle of DM Mice

**Figure 6:**
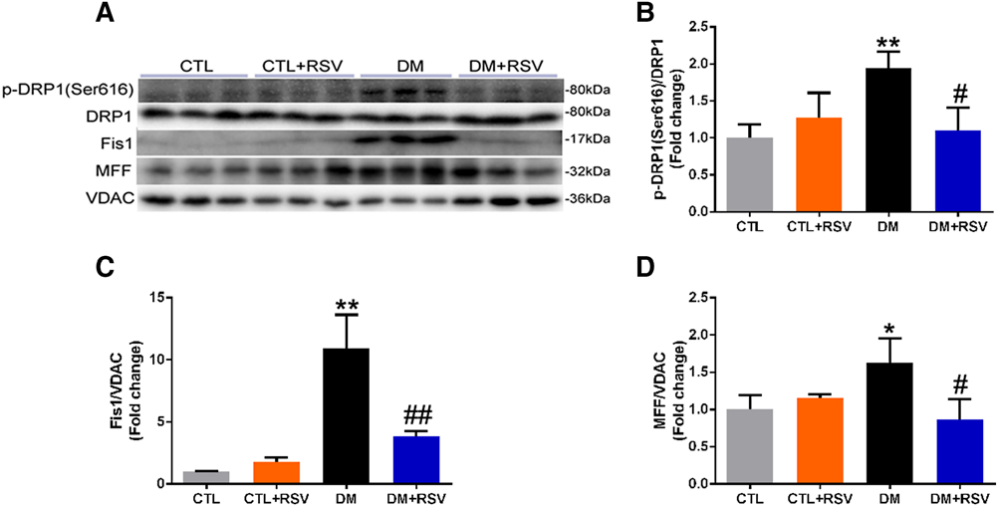
Feeding the diabetes mellitus (DM) mice with the RSV‐enriched diet prevented mitochondrial fission in the quadriceps muscle. A) Representative western blots using antibodies against phosphorylated dynamin‐related protein 1 (p‐DRP1, Ser616), DRP1, mitochondrial fission‐1 (Fis‐1), mitochondrial fission factor (MFF), and voltage‐dependent anion channel (VDAC). B) After quantification, the p‐DRP1 (Ser616)/DRP1, C) Fis‐1/VDAC, and D) MFF/VDAC ratios were calculated in mice fed either the control (CTL) or RSV‐enriched diet. The mitochondrial protein VDAC was used as a loading control. Data are expressed as fold change versus CTL and reported as mean ± SD, *n* = 4–6 per group; *p < 0.05; **p < 0.01 between CTL and DM groups. #*p* < 0.05; ##*p* < 0.01; ###*p* < 0.001 between DM and DM+RSV groups.

**Figure 7:**
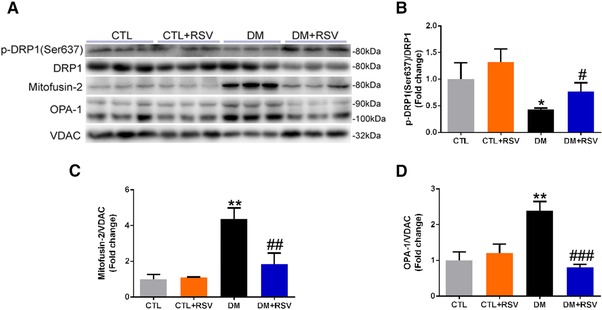
Feeding the diabetes mellitus (DM) mice with the RSV‐enriched diet prevented mitochondrial fusion in the quadriceps muscle. A) Representative western blots using antibodies against phosphorylated dynamin‐related protein 1 (p‐DRP1, Ser637), mitofusin‐2 (Mfn‐2), optic atrophy protein‐1 (OPA‐1), and voltage‐dependent anion channel (VDAC). After quantification, the B) p‐DRP1 (Ser637)/DRP1, C) Mfn‐2/VDAC, and D) OPA‐1/VDAC ratios were calculated in mice fed either the control (CTL) or RSV‐enriched diet. The mitochondrial protein VDAC was used as a loading control. Data are expressed as fold change versus CTL and reported as mean ± SD, *n* = 4–6 per group; *p < 0.05; **p < 0.01 between CTL and DM groups. #*p* < 0.05; ##*p* < 0.01; ###*p* < 0.001 between DM and DM+RSV groups.

We next investigated the effect of RSV on the levels of fission regulatory proteins (p‐Drp1(Ser616), Fis1, and MFF) and mitochondrial fusion regulatory proteins (p‐Drp1(Ser637), Mfn‐2, and OPA‐1) in the skeletal muscle of the DM mice (**Figures** 6A and 7A). Total Drp1 protein content was similar in the isolated mitochondria from all groups (Figures 6A and 7A). However, the phosphorylation of Drp1 (Ser616) which stimulates mitochondrial fission was increased in the DM group, and these changes appeared to be attenuated with RSV treatment (Figure 6B). Interestingly, Fis1 and MFF proteins were significantly higher in the isolated mitochondria from the DM group, and these increases were impeded by RSV (Figure 6C and D). In contrast, the phosphorylation of Drp1 (Ser637), which inhibits mitochondrial fission, was decreased in the DM group, and these changes appeared to be attenuated with RSV administration (Figure 7B). Both OPA‐1 and Mfn‐2 levels were upregulated in isolated mitochondria from the DM group, and this upregulation was hindered by RSV treatment (Figure 7C and D). These data suggest that mitochondrial fission and fusion were higher in diabetic muscle, and these processes were prevented by treating with RSV.

### 3.8 Resveratrol Prevents Muscle Mitophagy

**Figure 8:**
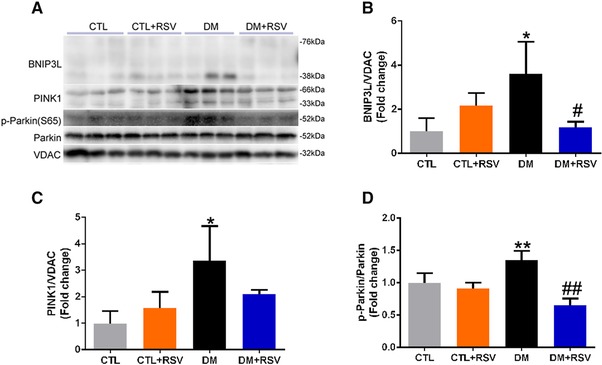
Feeding the diabetes mellitus (DM) mice with the RSV‐enriched diet inhibited mitophagy in the quadriceps muscle. A) Representative western blots using antibodies against NIP3‐like protein X (BNIP3L), PTEN‐induced putative kinase‐1 (PINK1), p‐Parkin, Parkin, and voltage‐dependent anion channel (VDAC). After quantification, the B) BNIP3L/VDAC, C) PINK‐1/VDAC, and D) p‐Parkin (S65)/Parkin ratios were calculated in mice fed either the control (CTL) or RSV‐enriched diet. The mitochondrial protein VDAC was used as a loading control. Data are expressed as fold change versus CTL and reported as mean ± SD, *n* = 4–6 per group; *p < 0.05; **p < 0.01 between CTL and DM groups. #*p* < 0.05; ##*p* < 0.01 between DM and DM+RSV groups.

We studied the effects of RSV on mitophagy markers (i.e., BNIP3L, PINK1, and Parkin) in isolated mitochondrial fractions (**Figure** 8A). Interestingly, the levels of BNIP3L and phosphorylated Parkin were significantly increased in the DM group and attenuated by RSV treatment (Figure 8B and D). However, the level of PINK1 in the DM group was increased, but only partially attenuated with RSV, as these differences did not reach statistical significance (Figure 8C).

## 4 Discussion

In the present study, we demonstrated that RSV supplementation enhances muscle mass and improves physical activity by increasing mitochondrial content and suppressing the activation of the ubiquitin–proteasome system (UPS), autophagy, and apoptosis in DM mice. Meanwhile, we demonstrated—for the first time—that RSV supplementation improves defective MQC by increasing mitochondrial biogenesis, suppressing the activation of mitophagy, and reducing mitochondrial fission and fusion in the skeletal muscle of DM mice.

The preservation of muscle mass in DM+RSV mice correlated with activation of the UPS, consistent with previous reports showing that RSV limited UPS activation and muscle atrophy under several catabolic conditions,^21^, ^22^, ^23^, ^24^, ^25^ including DM.^33^ In agreement with our findings, RSV only reduced, but did not fully suppress, elevated MuRF‐1 protein levels in diabetic muscle in these reports. The preservation of muscle mass and the higher induction of MuRF‐1 protein expression in DM mice are in accordance with a major role of MuRF‐1 in targeting major contractile proteins for breakdown by the 26S proteasome.^34^ In addition, the present findings indicate that RSV prevents elevated ubiquitin in diabetic muscle. We further showed that markers of autophagosome formation (LC3‐II) and apoptosis (cleaved caspase‐3) were induced to a lower level in RSV‐treated DM mice. We and others have already established the importance of autophagy and apoptosis in muscle atrophy,^27^, ^35^ but we report here for the first time that using an RSV‐supplemented diet can regulate autophagy and apoptosis in diabetic muscle. Collectively, the data suggest that RSV supplementation improves muscle mass by suppressing activation of the UPS, autophagy, and apoptosis in DM mice.

It has recently been shown that muscle mitochondrial content and function declines with DM, age, CKD, and disuse.^3^ In the present study, our observations suggest that the reduction in muscle mitochondria induced by DM is prevented by RSV treatment, which is consistent with previous reports that demonstrated that RSV increased muscle mitochondrial content and improved physical performance in metabolic syndrome models.^25^, ^36^ In addition, we demonstrated that mitochondrial biogenesis‐related proteins, such as PGC‐1α, NRF‐1, mtTFA, and Cox IV are significantly decreased in diabetic muscle and effectively rescued by RSV treatment; this is consistent with prior studies that have identified that mitochondrial biogenesis is significantly repressed in muscle from DM models, while mitochondrial biogenesis by RSV treatment ameliorates muscle atrophy.^37^ This corresponds to a previous report in which CKD resulted in decreased PGC‐1α, mtTFA, and ATP production leading to muscle atrophy.^38^ Muscle‐specific PGC1‐α transgenic mice are protected from the reductions in mitochondrial function and content observed during aging.^39^ PGC‐1α loss also results in reduced muscle mitochondrial content and ATP production.^40^, ^41^, ^42^ In addition, RSV‐induced activation of PGC‐1α occurs in a SIRT1‐dependent manner in skeletal muscle,^43^, ^44^ and leads to increased mitochondrial biogenesis. These data imply that RSV can increase muscle mitochondria by activating the PGC‐1α/NRF‐1/mtTFA signaling pathway in DM mice.

Mitophagy is critical for skeletal muscle function and removes damaged and dysfunctional mitochondria to maintain healthy ones. Impaired mitophagy leads to an accumulation of damaged mitochondria, which can negatively regulate muscle mass.^3^, ^45^, ^46^ It has been demonstrated that dysfunctional, depolarized mitochondria can produce higher amounts of ROS, leading to apoptosis and subsequent mitochondrial depolarization. Previous studies demonstrate that mitochondrial depolarization precedes mitophagy as a defensive response.^47^ One of our ongoing hypotheses is that mitophagy is activated by DM to eliminate the structurally and functionally damaged mitochondria in the atrophying muscle. Expectedly, the mitophagy‐related proteins, BNIP3L, PINK1, and Parkin, were all dramatically increased in diabetic muscle, which is consistent with previous studies in muscle atrophied in aging^48^ and immobilization models.^49^ Here, we hypothesized that RSV treatment contributed to adjustment of the rebalance between mitochondrial biogenesis and the removal of abnormal or damaged mitochondria by the mitophagy in DM muscle. Indeed, we show that RSV treatment decreased expression of several mitophagy protein markers associated with improved mitochondrial biogenesis. Interestingly, we showed that RSV supplementation suppresses the activated mitophagy in diabetic muscle atrophy. Overall, our results suggest that RSV supplementation improves mitochondrial function and suppressed the activation of mitophagy in diabetic muscle.

Balancing fission and fusion events is essential for proper maintenance of mitochondrial dynamics and function. Mitochondrial fission is necessary for skeletal muscle mitochondrial maintenance and quality.^50^ Accelerated fission results in pro‐apoptotic signals that lead to mitochondrial isolation from the network and reduction in the efficiency of ATP generation,^51^ which is often regarded as a sign of mitochondrial dysfunction.^52^, ^53^, ^54^ In contrast, failure to undergo fission will result in mitochondrial dysfunction and muscle atrophy.^9^, ^53^, ^54^ Interestingly, impairment of fission leads to the disruption of mitophagy, followed by the accumulation of damaged and dysfunctional organelles. In our study, markedly increased levels of the mitochondrial fission protein markers, p‐DRP1 (Ser637), MFF, and Fis1, were observed in diabetic muscle, which is consistent with previous studies in murine muscle atrophied by denervation or immobilization^9^, ^49^ and hindlimb suspension.^55^ However, damaged mitochondria can be repaired by fusion with healthy mitochondria, which allows the contents of healthy and dysfunctional mitochondria to be mixed. The loss of mitochondrial fusion has detrimental effects on skeletal muscle, as shown by the genetic knockout of Mfn‐1 and ‐2 resulting in muscle atrophy and reduced mitochondrial DNA.^56^ Moreover, OPA‐1 transgenic overexpression was sufficient to blunt denervation‐induced muscle atrophy and mitochondrial dysfunction.^57^ However, we previously reported that the fusion proteins, Mfn‐2 and OPA‐1, were substantially increased in diabetic muscle, which contrasted with the findings of previous reports.^58^, ^59^, ^60^ Quite interestingly, our current results show that RSV supplementation can suppress increased mitochondrial fission and fusion in diabetic muscle. An explanation for these differing results is not straightforward, because the components of the MQC system are closely interconnected, and an alteration in one system can impinge on the activation/inhibition of the other repair mechanism. Therefore, this interplay, which is of fundamental importance for the integration of mitochondrial function within the network, should be considered in future studies.

In conclusion, the present study shows that an 8‐week RSV supplementation preserves the body weight, muscle mass, mitochondrial content, and muscle function in DM mice. Furthermore, we report that RSV supplementation attenuates the DM‐induced alterations in the MQC processes, by increasing mitochondrial biogenesis, inhibiting the activation of mitophagy, and downregulating mitochondrial fission and fusion in the muscle of DM mice. These findings suggest a promising strategy, whereby improving MQC processes to increase mitochondrial content prevents and treats diabetic muscle atrophy.

## Conflict of Interest

The authors declare no conflict of interest.

## Supporting information

Supporting Information
