# Supplementary material for: Resveratrol Improves Muscle Atrophy by Modulating Mitochondrial Quality Control in STZ‐Induced Diabetic Mice
Source: Mol Nutr Food Res. 2018 Apr 23;62(9):1700941. doi: 10.1002/mnfr.201700941 (PMC6001753; doi:10.1002/mnfr.201700941)

**Supporting information**

Figure S1. Cytosolic and mitochondrial protein expression in isolated mitochondria and whole tissue lysate (A). Both isolated mitochondria and whole tissue lysates were immunoblotted with VDAC and GAPDH antibodies. The mitochondrial protein, VDAC, was evident in both samples; however GAPDH was only faintly evident in the isolated mitochondria. The absence of a prominent GAPDH band in the isolated mitochondria is indicative of little contamination from non-mitochondrial sources during the isolation procedure. (B) Electron microscopy micrographs (magnification: 12,000x) of isolated muscle mitochondria.


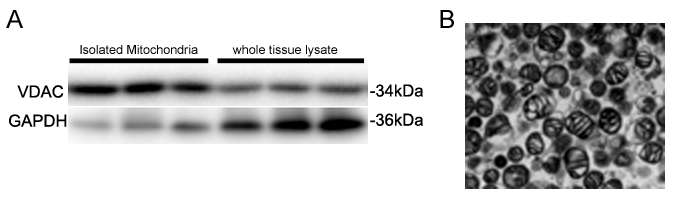

Supplement: Supplementary file 1 — Supporting Information [file MNFR-62-na-s001.docx]
